# Efficacy of radial shock wave therapy for ankle spasticity in patients with stroke within 3 months of onset: a prospective quasi-experimental study

**DOI:** 10.1186/s12984-026-01983-y

**Published:** 2026-05-03

**Authors:** Yuichi Hiramatsu, Hajime Yagura, Hiroaki Fujimoto, Megumi Hatakenaka, Teiji Kawano, Shota Segawa, Makiko Rai, Ryutaro Hayashi, Akihiko Nakae, Haruka Asano, Takuya Ogawa, Ichiro Miyai

**Affiliations:** 1 Neurorehabilitation Research Institute, Morinomiya Hospital, 2-1-88 Morinomiya, Joto-ku, Osaka, 536-0025 Japan; 2 Department of Rehabilitation, Toyama University Hospital, Toyama, Japan; 3 Department of Neurology, National Hospital Organization Osaka National Hospital, Osaka, Japan

**Keywords:** Modified ashworth scale, Passive range of motion, Poststroke spasticity, Radial shock wave, Subacute stroke

## Abstract

**Background:**

Extracorporeal shock wave therapy (ESWT) is widely used to reduce poststroke spasticity (PSS). However, limited evidence exists regarding its efficacy in patients with stroke within 3 months of onset. Therefore, this study aimed to investigate the association between ESWT administered 1 to 3 months after stroke and changes in spasticity and joint mobility in patients with PSS.

**Methods:**

Prospective quasi-experimental study. Fifty-two patients with PSS affecting the ankle joint were enrolled from March 2023 to March 2025, and allocated into three groups based on the time elapsed from stroke onset: 1, 2, or 3 months. All patients underwent radial ESWT to the gastrocnemius and soleus muscles once weekly for 3 consecutive weeks. Spasticity and joint mobility were evaluated using the Modified Ashworth Scale (MAS) and passive range of motion (PROM) measurements before and after each session, and at 1 and 5 weeks post-treatment.

**Results:**

Except for the MAS score obtained after the first session, significant immediate reductions in the MAS scores and PROM measurements were observed after all shock wave therapy sessions. Compared to baseline, cumulative changes were greatest after the third session, with a mean reduction of 0.6 points in the MAS score and a 6.4° increase in the PROM. These changes were maintained for 5 weeks. No serious adverse events related to shock wave therapy were reported.

**Conclusions:**

ESWT during the early subacute stage was associated with improvements in spasticity and joint mobility in patients with PSS. Repeated sessions showed greater cumulative changes compared with a single session.

*Trial registration* UMIN-CTR000050477.

## Background

Poststroke spasticity (PSS) is a common complication that adversely affects motor performance, functional independence, and quality of life [1]. Its management typically requires a comprehensive, multimodal approach that includes pharmacological and surgical treatments, physical and mechanical modalities, exercise-based rehabilitation, and orthotic interventions [1]. In addition to pharmacological treatments such as botulinum toxin type A (BoNT-A), several noninvasive physical modalities—including whole-body and focal vibration therapy, transcutaneous electrical nerve stimulation (TENS), and neuromuscular electrical stimulation (NMES)—have been investigated. Systematic reviews suggest that these approaches may reduce spasticity and improve joint mobility or motor outcomes when used as adjuncts to comprehensive neurorehabilitation programs, although heterogeneity in protocols and outcomes limits definitive conclusions [1].

Extracorporeal shock wave therapy (ESWT) has recently emerged as a noninvasive treatment for PSS [2], demonstrating efficacy and safety comparable to botulinum toxin therapy and ranking second in the accumulation of high-quality evidence [1, 3]. ESWT delivers acoustic energy to biological tissues through rapid alternations of positive and negative pressure [4]. The positive pressure phase concentrates mechanical stimulation at interfaces with differing acoustic impedance, while the negative pressure phase induces cavitation, generating secondary mechanical energy [5–7]. Through mechanotransduction and direct mechanical effects, ESWT may disrupt abnormal cross-linking in fibrotic or contracted tissues, thereby promoting structural remodeling and improving tissue extensibility [8–11].

The mechanisms underlying the effects of ESWT on spasticity are not fully understood. Proposed mechanisms include changes in muscle rheological properties, increased nitric oxide production, reduced motor neuron excitability, neuromuscular junction dysfunction, and improved microcirculation [3, 11, 12]. PSS comprises both neural components, associated with increased reflex-mediated muscle tone, and nonneural components, related to passive stiffness of connective tissues [13–15]. Current evidence suggests that ESWT primarily affects nonneural components, as most studies report improvements in muscle stiffness, elasticity, viscosity, and passive range of motion, with limited or no effects on neural excitability [9, 16–31].

Several issues remain unresolved regarding the optimal clinical use of ESWT for spasticity, including appropriate dosage, mechanisms of action, timing of intervention, and outcome selection. These questions are particularly relevant in the subacute phase after stroke, during which PSS typically emerges (7–90 days post-onset) and may progressively worsen [32, 33]. This period is also characterized by heightened neuroplasticity, during which rehabilitation interventions may have the greatest impact on recovery [34]. However, emerging spasticity during this phase may interfere with motor learning and functional recovery if not adequately managed [35].

Although botulinum toxin therapy is commonly used to treat PSS in the subacute phase [36–40], its effects on functional recovery remain unclear. ESWT has therefore gained attention as a potential noninvasive alternative or adjunct to BoNT-A. Evidence suggests that radial ESWT may enhance the effects of BoNT-A and is most effective when integrated into a comprehensive neurorehabilitation program rather than applied as a stand-alone intervention [1, 41]. Nevertheless, most existing studies on ESWT have focused on patients in the chronic phase (> 3 months post-stroke), and evidence regarding its efficacy in the subacute phase remains limited [41–44].

Accordingly, this study investigated whether early administration of radial ESWT could improve spasticity and joint mobility in patients within 3 months of stroke onset. We hypothesized that repeated ESWT sessions would produce cumulative therapeutic effects that persist beyond the treatment period. Outcomes were evaluated across three time points: immediate effects (before and after each session), short-term effects (baseline to after three sessions), and follow-up effects (1 and 5 weeks after the final session).

## Methods

### Study design

**Fig. 1:**
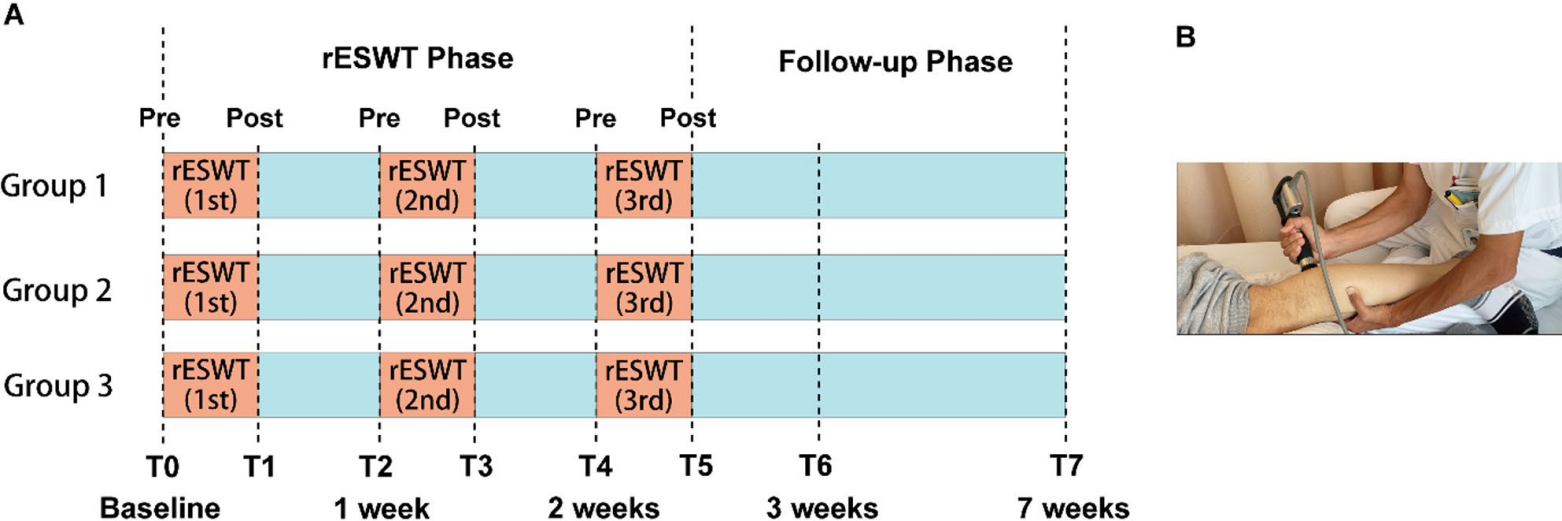
Study design and administration of ESWT on the gastrocnemius muscle. **A** Schematic representation of the study design. **B** Setup for radial ESWT. *Pre* before the therapy session, *Post* after the therapy session, *rESWT* radial extracorporeal shock wave therapy, *T* time point

This prospective, non-blinded, quasi-experimental study utilized a pre–post intervention, open-label, nonrandomized, and uncontrolled design. The study was conducted at [details omitted for double-anonymized peer review] (convalescent) rehabilitation ward at [details omitted for double-anonymized peer review] between March 2023 and March 2025. Patients were categorized into three groups based on the time that had elapsed at the time of enrollment since the onset of stroke: Groups 1, 2, and 3 included inpatients at approximately 1 (30 ± 10 days), 2 (60 ± 10 days), and 3 (90 ± 10 days) months after stroke, respectively. The major determinant of group allocation was the time between stroke onset and transfer from an acute hospital to the rehabilitation ward, which reduced potential selection bias despite the nonrandomized design. Patients underwent a conventional rehabilitation program comprising physical, occupational, and speech–language therapy, at least 5 days per week. The maximum duration of daily therapy was 180 min, including a minimum of 60 min of physical or occupational therapy. Following group allocation, clinical assessments were conducted eight times: before and after the first ESWT session (T0 and T1), before and after the second session (T2 and T3), before and after the third session (T4 and T5), 1 week after the final session (T6), and 5 weeks after the final session (T7; Fig. 1A). Each patient underwent three sessions of ESWT targeting the ankle plantar flexors (the gastrocnemius and soleus muscles), administered once weekly over a 3-week period. Outcome measures included the Modified Ashworth Scale (MAS) score and passive range of motion (PROM). Adverse events were assessed based on immediate posttreatment observations and a review of the medical records.

### Participants

Patients with stroke were recruited from those admitted to the rehabilitation ward. The inclusion criteria were as follows: (1) first-ever stroke, with unilateral cerebral hemorrhage or cerebral infarction confirmed using computed tomography or magnetic resonance imaging; (2) presence of hemiplegia; (3) within 3 months of stroke onset; and (4) a MAS score for the ankle of greater than 1. The exclusion criteria were as follows: (1) current treatment for spasticity (oral or injectable　therapies, including BoNT-A, vibration therapy, or electrical stimulation); (2) a Mini-Mental State Examination (MMSE) score of below 20; and (3) contraindications to ESWT, as defined by the International Society for Medical Shockwave Treatment [4, 5].

This study was reviewed and approved by the Research Ethics Board of Morinomiya Hospital (Approval No. 511, dated February 22, 2023) and was conducted in accordance with the Declaration of Helsinki. Written informed consent was obtained from all participants. The study was registered in the University Hospital Medical Information Network Clinical Trials Registry (trial identity UMIN-CTR000050477).

### Interventions

The Physio Shockmaster^®^ (Sakai Medical Co., Tokyo, Japan) was used to administer radial ESWT (Table 1). The treatment was performed with the patient in the supine position (Fig. 1B). The therapy parameters and administration methods were as follows: (1) the D-Actor applicator (20 mm) was applied to the entire belly of the muscle and myotendinous junction (2,500 shots, 1.6 bar, 17 Hz), and (2) the Beam applicator (15 mm) was used to target specific localized areas of the belly of the muscle or myotendinous junction (1,500 shots, 2.0 bar, 10 Hz). Based on the concept of motor point injections in botulinum toxin therapy, which is supported by both theoretical and some empirical evidence [42–45], the local administration sites of ESWT in this study were determined according to anatomical localization [46, 47].

Patients underwent a conventional rehabilitation program consisting of physical, occupational, and speech–language therapy, provided at least 5 days per week. Physical and occupational therapy were delivered for up to 1 h each per day, and speech–language therapy was provided as clinically indicated. The rehabilitation program included mobility and gait training, activities of daily living training, joint range-of-motion exercises, muscle strengthening, balance and endurance training, as well as speech, swallowing, and cognitive–linguistic interventions related to higher brain dysfunction (Table 2).

**Table 1:** rESWT treatment parameters and administration

Item	Description
Device	Physio Shockmaster^®^ (Sakai Medical Co., Tokyo, Japan)
Type of ESWT	Radial extracorporeal shock wave therapy
Treatment area	Gastrocnemius–soleus muscle complex (affected side)
Patient position	Supine position
Applicator 1	D-Actor applicator (20-mm diameter)
Target	Entire muscle belly and myotendinous junction
Parameters	2,500 shocks, 1.6 bar, 17 Hz
Applicator 2	Beam applicator (15-mm diameter)
Target	Localized areas of the muscle belly or myotendinous junction
Parameters	1,500 shocks, 2.0 bar, 10 Hz
Total number of shocks	4,000 shocks per session
Treatment frequency	Once per week
Total number of sessions	3 sessions
Site selection rationale	Based on anatomical localization and the concept of motor point injections used in botulinum toxin therapy

**Table 2:** Conventional rehabilitation program

Component	Description
Physical therapy	Mobility training, gait training, balance exercises, lower-limb strengthening, joint range-of-motion and stretching exercises, and endurance training
Occupational therapy	ADL training, upper-limb motor training, joint range-of-motion exercises, muscle strengthening, balance training during functional tasks, and fine motor skill training
Speech–language therapy	Speech and articulation exercises, swallowing rehabilitation, language and communication therapy, and cognitive–linguistic training related to higher brain dysfunction
Program delivery	Provided according to standard institutional stroke rehabilitation protocols
Program duration	Throughout the study period
Concomitant treatments	No additional anti-spastic interventions beyond routine rehabilitation

### Outcome measures

The outcome measures were the MAS score and PROM, because these are widely used and considered the gold standard clinical tools for assessing spasticity and joint mobility in patients with PSS. Outcomes were measured by a rehabilitation physician and physical therapist, with the patient in the supine position. The measurement criteria for the MAS were based on the report by Bohannon and Smith [48]. This scale consists of six grades: 0 (no increase in muscle tone); 1 (slight increase in muscle tone, manifested by a catch and release or by minimal resistance at the end of the range of motion when the affected part[s] is moved in flexion or extension); 1+ (slight increase in muscle tone, manifested by a catch followed by minimal resistance throughout the remainder [less than half] of the range of motion); 2 (more marked increase in muscle tone through most of the range of motion, but affected part[s] are easily moved); 3 (considerable increase in muscle tone, passive movement difficult); and 4 (affected part[s] rigid in flexion or extension). PROM was measured using an electronic goniometer.

### Statistical analysis

All statistical analyses were performed using R software version 4.5.1 [49]. Baseline differences among the three groups were assessed using the Kruskal–Wallis test for continuous variables and chi-square tests or Fisher’s exact tests for categorical variables, as appropriate. Two-tailed p-values of less than 0.05 were considered statistically significant. For clinical interpretability, descriptive statistics, including means and standard deviations of MAS scores and PROM measurements at each time point, are reported.

For the ordinal outcome (MAS score), longitudinal changes were assessed using a cumulative link mixed model with a logit link function, implemented with the ordinal package in R. The model included fixed effects for Time (eight levels: T0 to T7), Group (three levels: Group 1 to Group 3), and their interaction (Time × Group), as well as a random intercept for each participant to account for within-participant correlations. Post hoc pairwise comparisons of estimated marginal means were conducted for significant fixed effects only, using the emmeans package with Tukey-adjusted p-values to correct for multiple testing. Model estimates were exponentiated to obtain odds ratios, which were used as effect sizes, and presented with 95% confidence intervals and p-values. Although no universally accepted cutoffs exist for interpreting the magnitude of the odds ratio, values of approximately 1.68 are typically considered small effects, 3.47 are considered moderate effects, and values above 6.71 are considered large effects [50].

For the continuous outcome, PROM, longitudinal changes were analyzed using a linear mixed effects model implemented with the lme4 package in R. The model included fixed effects for Time (eight levels: T0 to T7), Group (three levels: Group 1 to Group 3), and their interaction (Time × Group), as well as a random intercept for each participant to account for within-participant correlations. Prior to model fitting, residual normality and homogeneity of variance were assessed using Q–Q plots and Levene’s test, respectively. Post hoc pairwise comparisons of estimated marginal means were performed only when fixed effects were significant, using the emmeans package with Tukey-adjusted p-values to correct for multiple comparisons. Effect sizes were calculated for key pairwise contrasts, such as changes from before to after the intervention, based on estimated marginal means and the residual standard deviation of the model [51]. Effect sizes were interpreted according to Cohen’s conventions: d = 0.2 (small), 0.5 (medium), and 0.8 (large).

## Results

### Participant characteristics

**Fig. 2:**
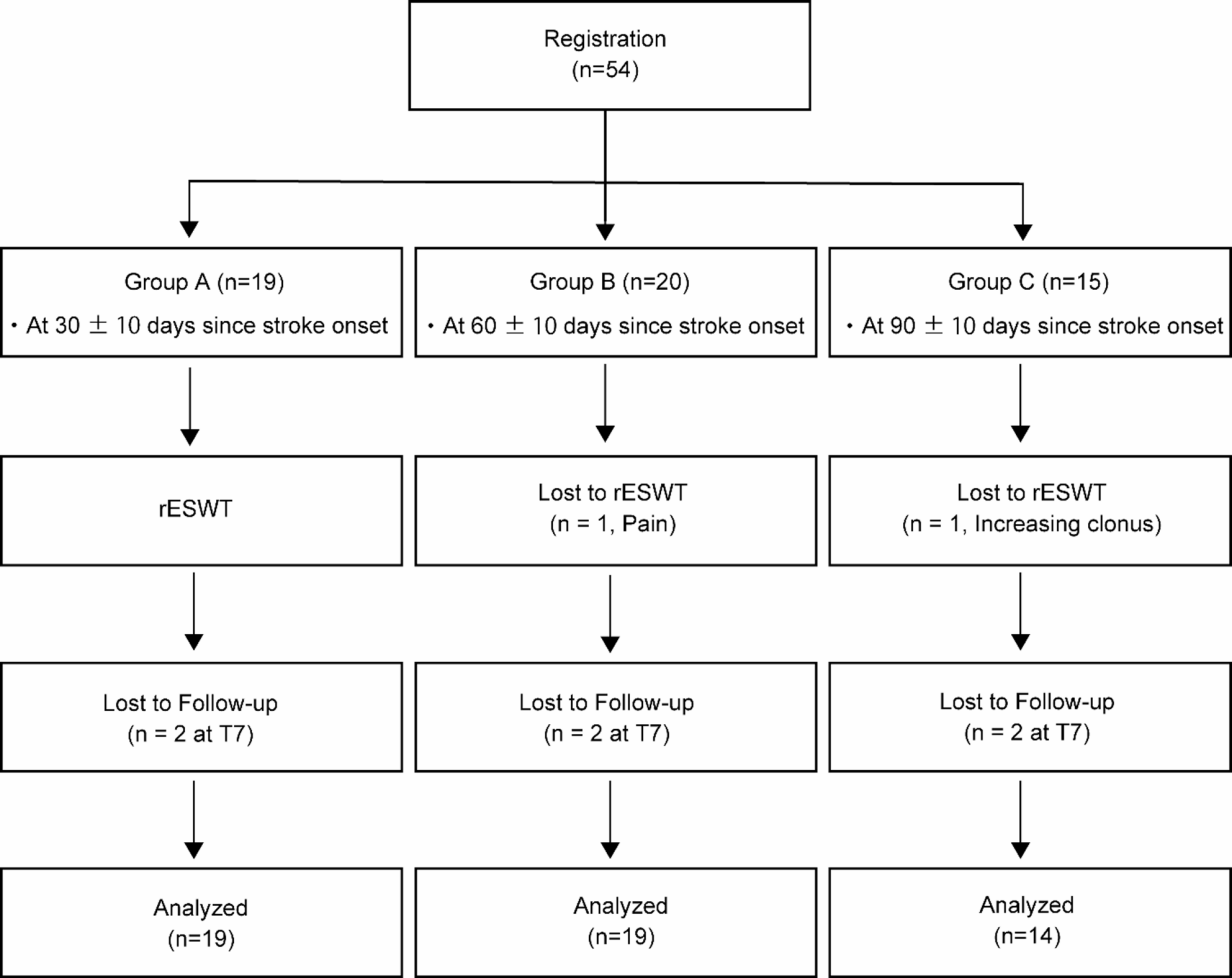
Flowchart of selection of study participants. *rESWT* radial extracorporeal shock wave therapy

Overall, 54 patients with stroke and PSS were treated at our institution between March 2023 and March 2025 and met this study’s eligibility criteria. A total of 52 patients assigned to the three groups (Group 1, *n* = 19; Group 2, *n* = 19; and Group 3, *n* = 14) completed the study (Fig. 2). Patient demographics and clinical characteristics are summarized in Table 3. No significant differences were observed among the three groups with respect to age, sex, stroke type, affected side, lesion location, cognitive function (MMSE), functional independence (FIM motor and cognitive scores), motor impairment assessed by the Fugl–Meyer Assessment (upper and lower extremities), sensory function (light touch and proprioception), spasticity severity (MAS), or ankle passive range of motion (PROM) (all *p* > 0.05). In contrast, disease duration differed significantly among groups (*p* < 0.001), reflecting the predefined grouping based on the timing of intervention after stroke onset. Two participants were excluded from radial ESWT due to pain or worsening clonus, and six additional participants were lost to follow-up after T7, which marked the end of the trial.

**Table 3:** Baseline clinical and demographic characteristics of participants

Characteristic	Group 1	Group 2	Group 3	Total	*P* value
Patients, n	19	19	14	52	
Age, years	56.9 (10.2)	60.9 (14.0)	51.6 (13.1)	57.0 (12.8)	0.10
Sex (female/male), n	14/5	10/9	9/5	33/19	0.40
Disease duration, days	34.6 (4.2)	61.1 (4.1)	92.4 (8.7)	59.8 (23.6)	0.01
Stroke type (hemorrhagic/ischemic, n)	5/14	6/13	4/10	15/37	1.00
Affected side (right/left), n	5/14	8/11	6/8	19/33	0.50
Lesion site (cortical/subcortical/brainstem/cortical + subcortical), n	1/16/1/1	0/14/1/4	0/10/0/4	1/40/2/9	0.38
Fugl–Meyer assessment motor score, points					
Upper limb	13.7 (13.6)	20.3 (17.5)	19.6 (18.6)	17.7 (16.4)	0.36
Lower limb	15.4 (8.4)	14.3 (6.0)	16.0 (8.4)	15.2 (7.5)	0.87
Fugl–Meyer assessment sensory score, points					
Upper limb	2.7 (1.3)	2.5 (1.1)	2.1 (1.7)	2.5 (1.3)	0.50
Lower limb	2.8 (1.2)	2.6 (1.2)	2.8 (1.1)	2.7 (1.1)	0.89
Fugl–Meyer assessment proprioception score, points					
Upper limb	4.3 (3.2)	4.0 (3.2)	3.4 (3.4)	3.9 (3.2)	0.71
Lower limb	5.5 (2.8)	5.8 (2.4)	5.1 (3.3)	5.5 (2.8)	0.97
Mini-Mental state examination score, points	27.7 (3.3)	25.4 (5.2)	23.7 (11.0)	25.8 (6.9)	0.28
Functional independence measure score, points					
Motor	44.8 (15.3)	44.6 (15.7)	52.3 (20.2)	46.8 (16.9)	0.31
Cognitive	26.6 (5.1)	25.1 (6.0)	24.6 (8.9)	25.5 (6.5)	0.66
Modified Ashworth Scale score (ankle), points	1.8 (0.8)	1.8 (0.6)	2.0 (0.7)	1.9 (0.7)	0.67
Passive range of motion (ankle), degrees	2.9 (9.4)	4.5 (9.3)	5.4 (7.6)	4.2 (8.8)	0.93

*UL* upper limb, *LL* lower limb

Values are expressed as mean (standard deviation)

### MAS outcomes

**Fig. 3:**
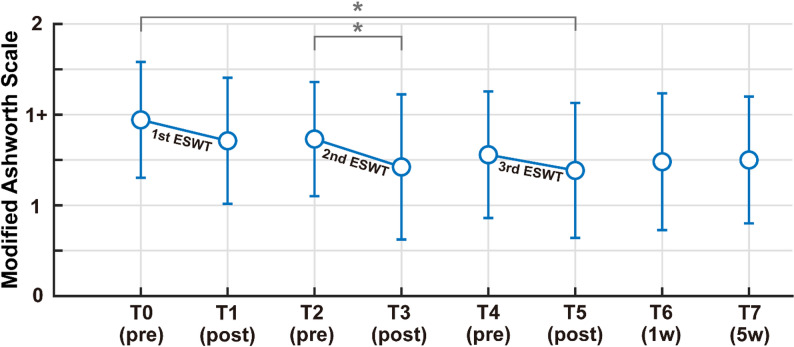
MAS scores at different time points. T0–T7 represent measurement time points from baseline to follow-up. *P < 0.05. ESWT, extracorporeal shock wave therapy; *pre* MAS score before ESWT session, *post* MAS score after ESWT session

Longitudinal changes in the MAS scores are presented in Fig. 3, with detailed estimates and comparisons shown in Table 4. A significant main effect of time was observed (χ²(7) = 67.98; *p* < 0.001), indicating changes across the eight time points. A decreasing trend in changes before and after each intervention was observed following the first and third interventions, although these changes did not reach statistical significance. A significant improvement was observed after the second intervention. During the 1-week intervals between interventions, only trends toward regression were observed, with no statistically significant changes. The greatest improvement was achieved after the third intervention, and this effect was maintained through the 5-week follow-up period. Compared to T0, the MAS score was significantly improved at all subsequent time points after the second intervention (T3–T7; all *p* < 0.001). Neither the main effect of the group (χ²(2) = 0.78; *p* = 0.678) nor the interaction between Group and Time (χ²(14) = 14.36; *p* = 0.423) was statistically significant.

Group-specific changes in MAS from baseline (T0) to post-intervention (T5) are summarized in Table 6. Mean reductions in MAS were observed in all groups, with changes of − 0.47 ± 0.61 in Group 1 (~ 1 month), − 0.37 ± 0.76 in Group 2 (~ 2 months), and − 0.29 ± 0.47 in Group 3 (~ 3 months). The corresponding within-group effect sizes ranged from moderate to large (Cohen’s dz = − 0.77, − 0.48, and − 0.61, respectively). Despite these descriptive differences, no statistically significant Group × Time interaction was detected in the mixed-effects model.

**Table 4:** Effect of ESWT on MAS scores

Time point	Mean (range)			Median (IQR)			OR	95% CI	*p*-Value
	Pre	Post	Diff	Pre	Post	Diff			
Immediate effects									
After 1st ESWT session (T0 vs. T1)	1.9 (0.6)	1.7 (0.7)	−0.2 (0.4)	2.0 (0.0)	2.0 (1.0)	0.0 (0.0)	4.29	[0.91, 20.28]	0.084
After 2nd ESWT session (T2 vs. T3)	1.7 (0.6)	1.4 (0.8)	−0.3 (0.5)	2.0 (1.0)	1.0 (1.0)	0.0 (1.0)	6.86	[1.45, 32.59]	0.004
After 3rd ESWT session (T4 vs. T5)	1.6 (0.7)	1.4 (0.7)	−0.2 (0.4)	2.0 (1.0)	1.5 (1.0)	0.0 (0.0)	2.71	[0.62, 11.84]	0.452
Short-term effects									
Overall effect after three ESWT sessions (T0 vs. T5)	1.9 (0.6)	1.4 (0.7)	−0.6 (0.6)	2.0 (0.0)	1.5 (1.0)	−0.5 (1.0)	30.42	[5.77, 160.36]	< 0.001
Follow-up effects									
1 week after 3rd ESWT session (T5 vs. T6)	1.4 (0.7)	1.5 (0.8)	0.1 (0.7)	1.5 (1.0)	2.0 (1.0)	0.0 (0.0)	0.59	[0.14, 2.50]	0.953
5 weeks after 3rd ESWT session (T5 vs. T7)	1.4 (0.7)	1.5 (0.7)	0.2 (0.7)	1.5 (1.0)	2.0 (1.0)	0.0 (0.0)	0.49	[0.10, 2.29]	0.856
Inter-session changes									
Post-1st vs. Pre-2nd ESWT (T1 vs. T2)	1.7 (0.7)	1.7 (0.6)	0.0 (0.4)	2.0 (1.0)	2.0 (1.0)	0.0 (0.0)	0.88	[0.19, 4.04]	1.000
Post-2nd vs. Pre-3rd ESWT (T3 vs. T4)	1.4 (0.8)	1.6 (0.7)	0.1 (0.6)	1.0 (1.0)	2.0 (1.0)	0.0 (0.5)	0.44	[0.10, 1.93]	0.691
Post-3rd ESWT vs. 1-week follow-up (T5 vs. T6)	1.4 (0.7)	1.5 (0.8)	0.1 (0.7)	1.5 (1.0)	2.0 (1.0)	0.0 (0.0)	0.59	[0.14, 2.50]	0.953

T0–T7 represent measurement time points from baseline to follow-up. Odds ratios (ORs) > 1 indicate a positive effect relative to the reference

*CI* confidence interval, *Diff* difference in MAS scores between ESWT sessions,* ESWT* extracorporeal shock wave therapy, *IQR* interquartile range, *OR* odds ratio, *Pre* MAS score before ESWT session, *Post* MAS score after ESWT session

### PROM

**Fig. 4:**
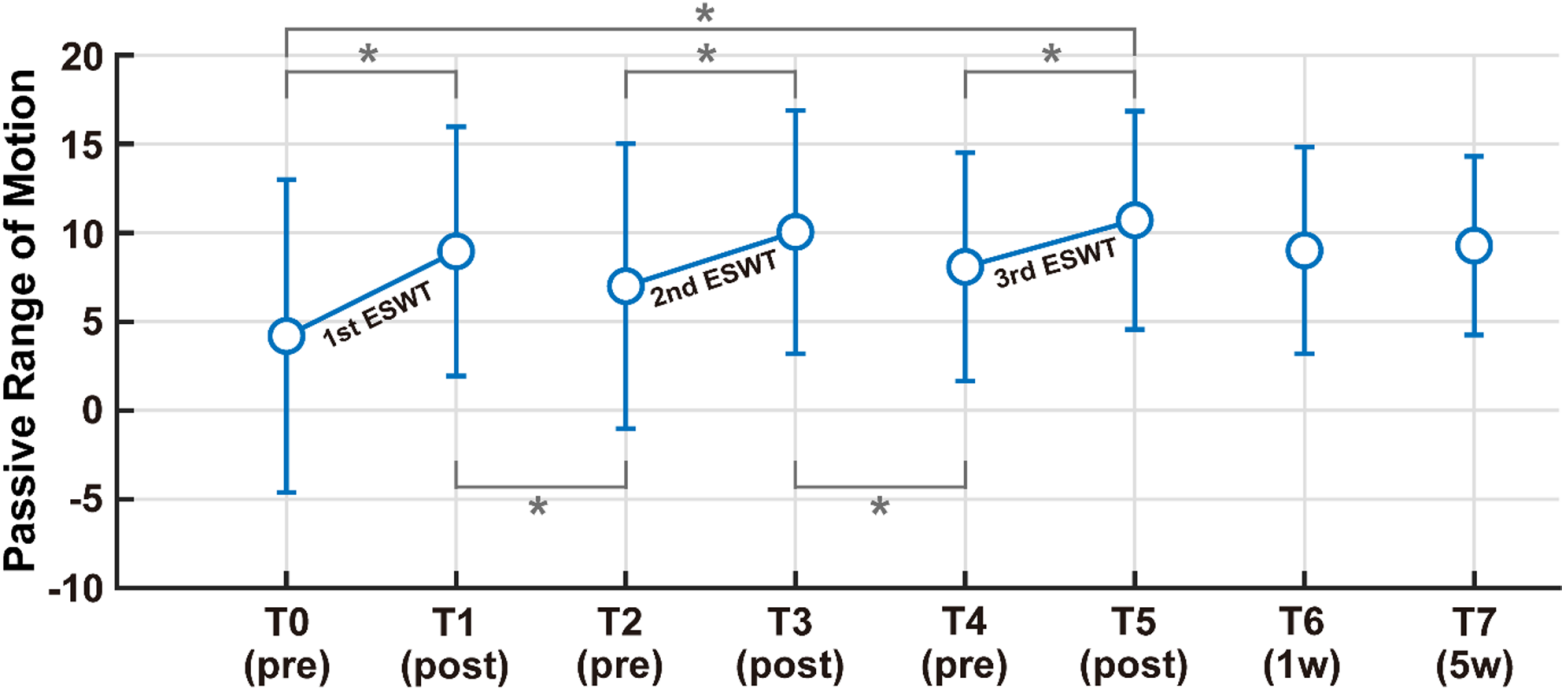
PROM at different time points. T0–T7 represent measurement time points from baseline to follow-up. Statistically significant differences were observed in immediate, inter-session, and short-term effects. *ESWT* extracorporeal shock wave therapy, *Pre* PROM before ESWT session, *Post* PROM after ESWT session

The longitudinal changes in PROM are presented in Fig. 4, with detailed estimates and comparisons shown in Table 5. A significant main effect of time was observed (F(7, 335) = 18.08; *p* < 0.001), indicating changes across the eight time points. At each phase, PROM measurements significantly improved following the intervention (T0 vs. T1, ES = 1.39; T2 vs. T3, ES = 0.93; T4 vs. T5, ES = 0.80). During the 1-week intervals between interventions, partial reductions in effect were observed (T1 vs. T2, ES = 0.61; T3 vs. T4, ES = 0.63). However, the greatest improvement was observed after the third intervention (T0 vs. T5, ES = 1.87), and this effect was maintained through the 5-week follow-up period (T5 vs. T6, ES = 0.33). Consequently, compared to baseline (T0), PROM scores remained significantly improved at all subsequent time points (T1–T7; all *p* < 0.005). The main effect of the group (F(2, 49) = 0.09; *p* = 0.917) and the Group × Time interaction (F(14, 335) = 1.39; *p* = 0.158) showed no statistical significance.

Group-specific changes in PROM from baseline (T0) to post-intervention (T5) are also presented in Table 6. PROM increased in all groups, with mean changes of + 5.71 ± 6.53 in Group 1, + 3.95 ± 5.62 in Group 2, and + 1.34 ± 5.32 in Group 3. The within-group effect sizes were large in Group 1 (dz = 0.88), moderate in Group 2 (dz = 0.70), and small in Group 3 (dz = 0.25). However, consistent with the primary analysis, the Group × Time interaction did not reach statistical significance.

### Adverse events

Mild adverse events were reported, including transient erythema, which resolved within a few hours. In a small number of cases, muscle contractions and joint movements were elicited while extracorporeal shock wave therapy was being administered. One participant experienced exacerbation of clonus, and another reported intolerable pain during or immediately after the first therapy session; both discontinued the intervention and were excluded from the study (Fig. 2).

**Table 5:** Effect of ESWT on PROM

Time point	Mean (range)			Median (IQR)			Estimate	95% CI	*p*-Value
	Pre	Post	Diff	Pre	Post	Diff			
Immediate effects									
After 1st ESWT session (T0 vs. T1)	4.2 (8.8)	9.0 (7.0)	4.8 (4.4)	4.7 (10.1)	9.4 (8.8)	4.3 (6.1)	−4.77	[− 6.84, − 2.69]	< 0.001
After 2nd ESWT session (T2 vs. T3)	7.0 (8.0)	10.0 (6.8)	3.1 (3.6)	7.2 (10.2)	10.2 (10.0)	1.9 (4.9)	−3.18	[− 5.26, − 1.11]	< 0.001
After 3rd ESWT session (T4 vs. T5)	8.1 (6.4)	10.7 (6.2)	2.6 (3.5)	8.2 (9.7)	10.8 (8.9)	2.5 (4.9)	−2.75	[− 4.82, − 0.67]	0.002
Short-term effects									
Overall effect after three ESWT sessions (T0 vs. T5)	4.2 (8.8)	10.7 (6.2)	6.5 (6.2)	4.7 (10.1)	10.8 (8.9)	5.0 (6.6)	−6.42	[− 8.49, − 4.34]	< 0.001
Follow-up effects									
1 week after 3rd ESWT session (T5 vs. T6)	10.7 (6.2)	9.0 (5.8)	−1.7 (3.5)	10.8 (8.9)	8.7 (8.8)	−1.9 (4.3)	1.76	[− 0.31, 3.84]	0.163
5 weeks after 3rd ESWT session (T5 vs. T7)	10.7 (6.2)	9.3 (5.0)	−1.1 (4.5)	10.8 (8.9)	8.9 (6.5)	−0.6 (4.9)	1.14	[− 1.07, 3.35]	0.765
Inter-session changes									
Post-1st vs. Pre-2nd ESWT (T1 vs. T2)	9.0 (7.0)	7.0 (8.0)	−2.0 (4.6)	9.4 (8.8)	7.2 (10.2)	−2.0 (4.9)	2.10	[0.03, 4.18]	0.044
Post-2nd vs. Pre-3rd ESWT (T3 vs. T4)	10.0 (6.8)	8.1 (6.4)	−2.0 (4.5)	10.2 (10.0)	8.2 (9.7)	−2.0 (6.1)	2.18	[0.10, 4.25]	0.032
Post-3rd ESWT vs. 1-week follow-up (T5 vs. T6)	10.7 (6.2)	9.0 (5.8)	−1.7 (3.5)	10.8 (8.9)	8.7 (8.8)	−1.9 (4.3)	1.76	[− 0.31, 3.84]	0.163

T0–T7 represent measurement time points from baseline to follow-up

*CI* confidence interval, *Diff* difference in PROM between ESWT sessions, *ESWT* extracorporeal shock wave therapy, *IQR* interquartile range, *Pre* PROM before ESWT session, *Post* PROM after ESWT session

**Table 6:** Group-specific mean changes and within-group effect sizes

Outcome	Group (time since stroke)	Mean ± SD (Baseline)	Mean ± SD (Post-intervention)	Mean change	Effect size (Cohen’s dz)
MAS	1 (~ 1 mo)	1.95 ± 0.71	1.47 ± 0.91	-0.47 ± 0.61	-0.77
	2 (~ 2 mo)	1.84 ± 0.60	1.47 ± 0.61	-0.37 ± 0.76	-0.48
	3 (~ 3 mo)	2.07 ± 0.62	1.79 ± 0.43	-0.29 ± 0.47	-0.61
PROM	1 (~ 1 mo)	2.91 ± 9.38	8.62 ± 6.04	+ 5.71 ± 6.53	0.88
	2 (~ 2 mo)	4.55 ± 9.34	8.50 ± 6.10	+ 3.95 ± 5.62	0.70
	3 (~ 3 mo)	5.45 ± 7.55	6.79 ± 7.57	+ 1.34 ± 5.32	0.25

Mean change represents within-group change from baseline (T0) to post-intervention (T5). Cohen’s d values indicate within-group effect sizes (Cohen’s dz). No statistically significant Group × Time interaction was observed in the mixed-effects models.* mo* months since stroke onset

## Discussion

Herein, we observed that ESWT was associated with improvements in both the MAS score and PROM in patients with PSS when administered within 1 to 3 months after stroke onset. Specifically, (1) immediate (single session) and short-term (once weekly for 3 weeks) ESWT were associated with improvements in the MAS score and PROM, and (2) these benefits were maintained for up to 5 weeks after the final session. These findings are consistent with previous reports on the efficacy of ESWT in patients with chronic-phase PSS, including immediate, short-term, and long-term effects [9, 16–29, 52].

It is important to distinguish between the immediate (pre–post session) effects and longer-term changes observed in this study. The immediate improvements in MAS and PROM observed after each ESWT session may reflect acute physiological responses, such as temporary changes in muscle viscoelasticity. In contrast, the cumulative improvements observed after three sessions and their maintenance through the 5-week follow-up period suggest more persistent changes. However,

interpreting these longer-term effects requires careful consideration of the study’s methodological limitations, particularly the absence of a control group and the confounding influence of spontaneous neurological recovery during the subacute phase (see Limitations section).

To date, only a few studies have examined the effectiveness of ESWT for PSS during the subacute phase. One such study, by Tirbisch (2015), targeted patients with subacute stroke between 3 weeks and 6 months after onset, with a mean time since stroke of approximately 4 months: Tirbisch found that ESWT demonstrated no significant superiority over conventional physiotherapy in reducing spasticity of the gastrocnemius muscles [30]. This absence of between-group significance may be attributed to the limited statistical power resulting from the small sample size. Nevertheless, a significant within-group reduction in spasticity, as measured by the MAS, was observed in the group that underwent radial ESWT. These findings suggest that radial ESWT may exert beneficial effects on PSS, an effect that may become more evident in future studies with larger sample sizes and adequate statistical power. A study by Moon et al. targeted patients with subacute stroke with spasticity of the ankle plantar flexor [31]. In that study, the mean time from stroke onset was approximately 2.7 months, and the effects of three ESWT sessions administered weekly over 3 weeks were investigated. The study findings showed that spasticity, assessed both clinically and biomechanically, was significantly reduced immediately and 1 week after treatment, although the effect was no longer statistically significant at 4 weeks after treatment [31]. The present study builds upon previous research and shows that administering ESWT for PSS during the subacute phase—within 1 to 3 months after stroke onset, a critical period of poststroke motor recovery [34]—was associated with immediate and short-term improvement in both the MAS score and PROM, potentially having implications for the early management of PSS. These findings are also consistent with recent studies on radial ESWT for ankle plantar flexor spasticity, most of which were conducted in patients with chronic stroke, reporting improvements in MAS and ankle PROM and suggesting the potential importance of treatment parameters such as session frequency and dosage [12, 24, 26].

The mechanism by which ESWT improves the MAS score and PROM in PSS has not been fully elucidated [11, 12]. In the current study, PROM was assessed based on R2 of the Modified Tardieu Scale (MTS); this scale reflects the nonreflex component and represents the maximal joint angle achieved when the limb is passively moved as slowly as possible [53]. In other words, PROM was intended to measure the pure joint range of motion after the resistance caused by the stretch reflex had disappeared. The MAS and MTS–Catch angle have been reported to correlate with PROM [54]. This suggests that, to some extent, changes in the MAS score involve changes in the nonreflex components of spasticity. However, PROM in the present study was used as a surrogate measure and does not allow direct or objective differentiation between neural and non-neural components of spasticity, as no biomechanical or neurophysiological assessments were performed. Accordingly, interpretations regarding the predominant effects of ESWT on nonreflex (viscoelastic) components should be regarded as hypothesis-generating rather than confirmatory.

Recently, joint torque has been reported to break down into neural, elastic, and viscous components during passive movement [55]. Administering BoNT-A primarily improves the neural (reflex) component [56], whereas ESWT mainly affects the viscoelastic (nonreflex) component [29]. However, basic research has recently indicated that ESWT may reduce compound muscle action potentials by destroying acetylcholine receptors at the neuromuscular junction [57–59]. Whether ESWT clinically decreases compound muscle action potentials remains unclear [9, 16–19]. Given that the early subacute poststroke period is characterized by active neural recovery and motor relearning, the potential neural effects of ESWT and their clinical implications should be interpreted with particular caution. Therefore, while the effects of ESWT are considered to predominantly influence the nonreflex components of spasticity, the potential for transient neuromuscular disruption during active neural recovery raises the question of whether ESWT could inadvertently interfere with motor relearning, warranting further investigation with functional outcome measures.

In the present study, repeated weekly sessions of ESWT resulted in cumulative improvements in both the MAS score and PROM. In contrast, when ESWT was applied at a frequency of once per week, the therapeutic gains in PROM significantly declined, suggesting that treatment frequency plays an important role in sustaining its effects. Previous reports have also indicated that three sessions of ESWT yield greater improvements and longer-lasting effects than a single session [60]. With respect to treatment dose, a dose-dependent effect was observed only in the group that received 4,000 shots—twice the amount administered to the control group—for ankle plantar flexor spasticity [25]. The optimal dosage for ESWT has not yet been clearly established [61]. In the present study, a total of 4,000 shock waves per session were delivered using combined applicators. This dosage was selected based on previously reported protocols for ankle plantar flexor spasticity after stroke, in which a range of 1,500 to 4,000 shots per session has been used in clinical studies [16, 17, 24, 26, 31]. Although several reports have suggested dose-dependent effects under specific stimulation conditions [25], the dose–response relationship of ESWT remains incompletely defined and appears to depend on multiple factors, including energy density, treatment frequency, and target tissue characteristics [61]. Therefore, the selected dosage was based on existing clinical practice rather than a definitive dose–response framework. These insights are valuable for the future optimization of therapy treatment protocols.

Although the Group × Time interaction was not statistically significant, descriptive patterns in effect sizes may offer insights into optimal timing (Table 6). For PROM, patients treated at approximately 1 month post-stroke (Group 1) showed large effects (Cohen’s dz = 0.88), compared to moderate effects at 2 months (dz = 0.70) and small effects at 3 months (dz = 0.25). This trend suggests that earlier intervention within the subacute phase may yield greater improvements in joint mobility. However, MAS effect sizes were more variable across groups. While these findings require confirmation in larger studies, they suggest that the timing of ESWT may influence treatment response.

Rarely have serious adverse events related to ESWT been reported. Previous studies have described only minor, transient adverse events, such as pain, muscle weakness, petechiae, and small blisters, which typically resolve within a few days [12]. Similarly, in the present study, mild adverse events such as erythema were observed, but these resolved spontaneously within a few hours. However, muscle contractions and joint movements were induced in a small number of patients while ESWT was being administered. These joint movements occurred periodically in accordance with the stimulation frequency, suggesting that repeated muscle contractions were triggered via tendon reflexes. To our knowledge, ESWT-induced muscle contractions or rhythmic joint movements have not been previously reported. Therefore, the transient exacerbation of clonus observed immediately after ESWT, along with these phenomena, may represent preliminary findings in this field.

From a clinical perspective, the magnitude and relevance of the observed changes warrant careful interpretation. Although statistically significant reductions in MAS (mean reduction of approximately 0.6 points) were observed, the MAS is an ordinal scale with known limitations in sensitivity and responsiveness [15, 48]. Moreover, minimal clinically important difference values for spasticity outcomes, particularly those based on the MAS, have been proposed mainly in chronic stroke populations and specific treatment contexts, such as botulinum toxin therapy, but remain inconsistent across studies and are not well established for the subacute poststroke period [15]. Therefore, the clinical meaningfulness of the observed MAS changes should be interpreted cautiously. In contrast, improvements in PROM may have practical implications for positioning, ease of care, and prevention of secondary musculoskeletal complications [8], even in the absence of direct functional outcome measures. However, because functional outcomes such as gait performance, balance, or activity-level measures were not assessed, it remains unclear whether reductions in spasticity and increases in joint mobility translated into meaningful functional recovery during the subacute phase. Future studies incorporating functional endpoints are needed to clarify the role of early ESWT as an adjunct to motor learning and functional rehabilitation [34].

### Limitations

Despite these generally favorable findings, some methodological limitations should be acknowledged. The non-randomized, non-blinded design without a control group may have introduced bias and residual confounding. Given that the intervention was conducted during the subacute poststroke period—when spontaneous neurological recovery is pronounced—the extent to which improvements can be attributed specifically to ESWT rather than natural recovery remains uncertain. Group allocation based on transfer timing does not fully control for confounders such as disease severity, recovery rate, or concurrent motor improvements.

The study relied exclusively on examiner-dependent clinical measures (MAS and PROM) susceptible to measurement variability in a non-blinded design. The absence of objective biomechanical or neurophysiological assessments and inter- or intra-rater reliability data limits measurement robustness and mechanistic interpretation. Although PROM was assessed based on R2 of the Modified Tardieu Scale to reflect nonreflex components, the inclusion of Modified Tardieu Scale catch angle (R1) as an additional outcome measure would have provided a more comprehensive assessment of spasticity components. Moreover, no statistical analysis was conducted to examine whether changes in MAS were associated with changes in PROM, limiting our understanding of the relationships between these outcomes. The absence of functional outcome measures further restricts the interpretation of how improvements translate into meaningful functional recovery.

Additionally, patients in the subacute phase represent a heterogeneous population regarding lesion characteristics and recovery trajectories, potentially influencing individual responsiveness. The study focused exclusively on ankle plantar flexor spasticity and cannot be generalized to other muscle groups or upper limb spasticity. The modest sample size and short follow-up (5 weeks) further restrict conclusions regarding longer-term efficacy.

### Future directions

Future studies with larger sample sizes, longer follow-up periods, and stratified analyses are needed to further clarify patient characteristics associated with optimal treatment response. Importantly, well-designed randomized controlled trials with appropriate sham or active control groups are required to confirm the true efficacy of ESWT, determine optimal treatment parameters, and establish its role in the management of spasticity during the subacute poststroke phase.

## Conclusions

Early administration of radial ESWT for PSS in the subacute phase was associated with immediate and cumulative improvements in the MAS score and PROM. These improvements were maintained for several weeks. These findings offer preliminary evidence suggesting the potential utility of radial ESWT as a safe, noninvasive early intervention for PSS. Further randomized controlled studies with larger samples and longer follow-up times are needed to validate and expand upon these results.

## Data Availability

The datasets generated during and/or analyzed during the current study are available from the corresponding author on reasonable request.
